# Family-centred HIV interventions: lessons from the field of parental depression

**DOI:** 10.1186/1758-2652-13-S2-S9

**Published:** 2010-06-23

**Authors:** Mark Tomlinson

**Affiliations:** 1 Department of Psychology, Stellenbosch University, Stellenbosch, South Africa

## Abstract

Traditionally, HIV prevention focuses on individual behaviours that place one at risk for HIV infection. Less widely regarded as a fundamental public health issue is parental depression and the detrimental effects it exerts on infant and child development, as well as its key contribution to non-fatal burden. Much like many HIV prevention and treatment interventions, programmes for depression focus almost exclusively on individuals and individual behaviour. This paper will use the extensive evidence base from research into parental depression as a model to argue for a family based approach to HIV prevention and treatment. The aim of this will be to make a case for targeting a broader set of behaviours that occur within families when developing and implementing interventions.

## Introduction

As a crucial public health problem, HIV/AIDS offers a stark challenge to dominant models of health promotion and prevention. Traditionally, HIV prevention focuses on individual behaviours that place one at risk for HIV infection. Less widely regarded as a fundamental public health issue is parental depression and the detrimental effects it exerts on infant and child development, as well as its key contribution to non-fatal burden. Much like many HIV prevention and treatment interventions, programmes for depression focus almost exclusively on individuals and individual behaviour.

Claeson and Waldman have argued for a move from disease-specific to people-specific interventions through promoting a limited set of household behaviours directly linked to the prevention and cure of common childhood illnesses [1]. This paper will use the extensive evidence base from research into parental depression as a model to argue for a family-based approach to HIV prevention and treatment. This will take the important person-centred approach of Claeson and Waldman one step further to include other family members and the interactions between them. In so doing, it argues for a paradigm shift in the treatment and prevention of HIV to one of a family-based approach in order to promote better child outcomes.

### Depression

In the most recent analysis by the "Countdown to 2015"collaboration, only 16 of the 68 priority countries that accounted for 97% of maternal and child deaths in 2005 were on track to meet targets for Millennium Development Goals 4 and 5 to reduce maternal and child mortality[2].

A key contributor to child wellbeing, which has been largely neglected in the broader discussion of maternal and child health, is the issue of mental health. Depression is the largest cause of non-fatal burden and the fourth leading cause of disease burden [3]; in many countries, it is the leading cause [4]. Mental disorders are not only linked to many other health conditions, but are also among the most costly medical disorders in terms of projected health care expenditure needed to treat them [5]. There are, however, significant barriers to care, with up to 70% of people with mental disorders never receiving any kind of care [6].

In the World Health Organization (WHO) World Mental Health survey, prevalence rates for any mood disorder ranged from 3.3% in Nigeria to 21.4% in the USA, while projected lifetime risk for any mood disorder ranged from 7.3% in China to 31.4% in the USA [7]. Depression is often co-morbid with other health conditions, such as diabetes, which in the case of South Africa, affects 2.6 million people and was the sixth leading cause of natural death in 2005 [8].

### Impact of depression on infants and children

Depression is a multi-generational disorder in that its psychological, social, biological and social consequences are felt by all members of the family and not solely by the person who is depressed [9]. This is particularly the case for children, but the impact of depression on other adult family members is also a concern [9]. Depression has been shown to affect social and leisure activities [10], to increase marital discord and conflict within families of depressed women [11], to be associated with heightened financial problems within families [10], and to increase demoralization in the non-depressed parent [12]; it also has a detrimental impact on the partners' own mental health [10]. In this way, depression is similar to HIV with regard to its effects on the broader family network.

### Physical development

The bulk of research on the impact of maternal mood on child development has focused on psychological, rather than physical, development, probably because most research has taken place in developed countries, where physical growth is not an area of particular concern. Cooper and colleagues [13] have, for example, shown in a British sample how postpartum depression can interfere with the mother's feeding of her infant.

The chief focus of this work, however, is on interactional issues, rather than on the implications of feeding problems for physical growth. Physical growth is, however, a major concern in developing countries, and the question arises as to whether this may be affected by maternal mood. In a study of low-income women in Goa, India, the presence of maternal depression in the post-partum period was found to be significantly associated with low infant weight and with shorter infant length at six months [14].

Rahman and colleagues [15] found that in rural Pakistan, infants of mothers depressed in the prenatal and the postnatal period showed growth retardation at several time points in the first year of life. In addition, chronic depression carried a greater risk for poor outcome than did episodic depression, while maternal mental state was associated with a higher risk of diarrhoea in infants. Based on these data, it has been estimated that the incidence of infant stunting in rural Pakistan would be reduced by 30% if maternal depression was eliminated from this population [6].

Rahman outlines a number of mechanisms that link depression to physical morbidity [6]. These include poor self-care skills, poor illness detection and poor care-seeking behaviour. In addition, as a result of the social with drawal that is characteristic of depressed women, they are more likely to receive inadequate antenatal care[16]. There is also an increased risk of poor fetal growth, premature birth and low birth weight among antenatally depressed women [17,18]; depression is also associated with riskier lifestyles, such as poor diet and smoking [19]. Rahman makes the important point that in low- and middle-income countries, environments are hostile and caregivers need to be vigilant of potential dangers to their infants and children [6]. So, for instance, high maternal responsiveness to a malnourished child's need for food and comfort has a direct positive impact on child growth[6].

### Socio-emotional development

An important question in seeking to understand the development of children growing up in conditions prevailing in low- and middle-income countries concerns the nature of the parenting that is possible under conditions of pervasive adversity. Preoccupation with external problems (e.g., poverty, lack of partner support), as well as more immediate difficulties (e.g., trauma and loss), may directly affect the parent's capacity to be responsive to his or her child. This difficulty may be further compounded by maternal mental health problems and, in particular, by the occurrence of depression.

Depression in the postpartum period has been found to affect between 10% and 15% of women in high-income countries [20], while rates in low- and middle-income countries have ranged from 23% in India [14] to 28% in Pakistan [15] and 34.7% in South Africa [21]. A large body of research evidence has implicated such depression in disturbances in the early mother-infant relationship and in compromised child development [22].

Depression in the postpartum period is particularly important in that the emerging processes of self and mutual regulation and social capacities make infants particularly vulnerable to early disruptions to interactions with their caregivers. Infants are born as social creatures primed for interaction with others [23]; infants are able to imitate facial expressions in the first hour after birth [24] and prefer their mothers' faces to those of strangers [25]. By three months of age, the capacities of the infant are even more sophisticated, having developed the ability to engage in complex turn-taking in interaction with an interactive partner [26].

In a South African study, depressed mothers were significantly less sensitive (more remote and more intrusive) in interaction with their infants in early face-to-face interactions than were non-depressed mothers, and infants of depressed mothers were also less positively engaged with their mothers [21]. These findings are consistent with those of several studies from low- and middle-income countries that have demonstrated how maternal depression results in less optimal maternal behaviours, such as unresponsiveness, insensitivity, intrusiveness and a lowered ability to assist infant affect regulation [26,27].

One of the consequences of such disturbances in the mother-infant relationship is an irritable and withdrawn infant, who may be more likely to develop an insecure attachment to his or her remote or intrusive mother [27,28]. Longitudinal research has found a raised rate of insecure infant attachment, impaired cognitive development, specifically in boys, and an elevated rate of behavioural and emotional problems in children of mothers with postpartum depression [22].

Stein reported increased anger and less affective sharing [29], while Murray found an increased level of behaviour problems in infants of depressed mothers [30]. In the South African study, children of depressed mothers were more likely to be insecurely attached at 18 months [31]. In the same study, maternal intrusive-coercive behaviour and remote-disengagement at two months, and sensitivity at 18 months, predicted infant attachment security [31].

### Depression and HIV as ports of entry for intervention

Given the high prevalence rates and disease burden of depression, key interventions have attempted to use depression as the port of entry into a family. Using depression as the port of entry is not without its complexities in that most people do not have access to the mental health system in order to be diagnosed with mental health problems. For example, in China, as few as 8% of people with mental health disorders seek professional help [32].

A key problem then is how to target interventions for depression as populations at high risk for depression remain difficult to identify [33]. One approach has been to use screening instruments, but their specificity is poor [34]. When depression has been successfully identified, there are a number of successful interventions that have been developed to treat it. Many of these interventions (although focused on the depression, either pharmacologically or behaviourally) have included, as one of their aims, the mitigation of the impact of the depression on the infant and the child.

An important finding in this regard has been that in some cases, even when the depression has been successfully treated, parenting quality does not necessarily improve [9]. If the aim of these interventions is the depression itself without a focus on the child (or when no children are present), this is not a problem. If the focus, however, is on the mitigation of the impact on children and the family, these data have important implications for where interventions should be targeted.

HIV is also commonly used as the port of entry into a family. One of the difficulties with this (and this is true of depression as well) is that it is a highly stigmatized disease. Rotheram-Borus and colleagues [35] have argued that using family wellness as the port of entry into the family will not only effectively combat HIV, but will also simultaneously avoid a narrow focus on sexual behaviour (that leads to stigma).

Another limitation of a narrow focus on depression or HIV as the port of entry is that the intervention fails to account for the fact that depression and HIV are exacerbated by problems in interpersonal relationships [36] and embedded in social and familial contexts characterized by substance abuse [14] and domestic violence [37]. Both HIV and depression form part of a constellation of other risk factors [9] frequently overlooked when the narrow focus is on HIV or depression.

### Treatment and prevention of depression

There is a considerable evidence base from high-income countries for the treatment of depression, both for anti-depressant pharmacotherapy and for a variety of inter-personal- and cognitive behaviour-based psychotherapeutic interventions. The evidence base from low- and middle-income countries is less extensive. A randomized trial conducted in India showed a benefit of anti-depressants over placebo [38], while a trial in Pakistan by Rahman and colleagues showed the effectiveness of a cognitive behavioural therapy (CBT)-based programme delivered by women health workers [39]. There is also evidence of the benefits of structured group CBT programme in Chile [40], and the effectiveness of group inter-personal psychotherapy in rural Ugandan villages [41].

Another approach has been to develop interventions that prevent depression. A number of psychosocial preventive interventions have been implemented (mostly in high-income countries), but evidence of effectiveness is limited. Dennis and Creedy [42] conducted a meta-analysis of psychological/psychosocial interventions that specifically targeted depression during the postpartum period, and found no preventive effect.

In the light of this lack of success of preventive interventions, an alternative approach has been to design interventions that improve the mother-infant relationship or parenting skills without directly targeting the depression. The rationale for this is to try and mitigate the impact of the postpartum depression during infancy, a highly vulnerable period for the infant. These approaches have been more promising, with benefits to parenting and the mother-infant relationship without an accompanying effect on maternal mood [9,43]. Targeting the effects of a particular disease (rather than the disease itself) is an intriguing idea, with implications for the prevention and treatment of a host of health conditions in low- and middle-income countries.

### Individual- and disease-focused interventions

#### Focus on the individual

HIV/AIDS offers a stark challenge to dominant models of the role of psychology in health promotion and prevention. Traditionally, HIV prevention focuses on individual behaviours that place one at risk for HIV infection. Models of health-promoting behaviours, such as the Theory of Reasoned Action [44] or the Health Belief Model [45], to name just two, have been used to try to understand individual behaviours and decision making that leads to HIV risk. HIV prevention programmes that draw on these models may have a primary aim of changing the factors that cause individuals to make the risk-taking decisions that they do. This is often achieved, for example, through education about health risk and protective behaviours, providing choices that aid decision making, and perhaps addressing some of the social factors, for example, the effects of stigma, that may influence individuals' behaviours and decisions.

The traditional health psychology approach has been vulnerable to criticism for its consistent focus on the individual as the unit of analysis and intervention. For example, Campbell [46] has argued that the utility of traditional models of health psychology in explaining complex behaviour and informing interventions is limited as they: (1) focus mainly on proximal determinants of behaviour, such as behavioural intentions and perceived norms; (2) often fail to show how these proximal determinants are determined by contextual realities; and (3) offer insight into which individual cognitive factors are related to health behaviours, but do not adequately provide guidance on how to change these cognitive factors.

Depression interventions often involve the targeting of a particular family member (the "depressed person") with little understanding of, sensitivity to, or interventions directed at how the depression may be determined by contextual realities.

A family-based approach requires us to question the notion that it is the rational intentions of individuals that are the key to health behaviour outcomes. We need to understand the degree to which these intentions are not only constrained by, but also shaped by, broader social factors, such as socio-economic factors and issues of power relations, including gender relations. Safe sex, to give a key example, is only marginally an issue of individual choice or reasoned action in a context within which risky sexual encounters that are detrimental in the long term may constitute the only available means of gaining access in the short term to food and money, and to avoiding violence and physical abuse. Finally, focusing on the individual, rather than the family, is not only less preferable, but in fact creates problems, such as when women are identified as HIV+ before their partners and families often resulting in them being blamed with subsequent stigma, exclusion and, in many cases, violence [47].

#### Focus on the disease

Claeson and Waldman [1] have convincingly argued that significant gains in child survival and improvements in child health will depend to an increasing degree on what happens in the household, in combination with a responsive and supportive health system. They go on to argue that there should be a focus on the promotion of a limited number of household behaviours that have a direct link to childhood illness.

Traditionally, a narrow disease-focused model has dominated health interventions. For example, the primary aim of most interventions that target pregnant, HIV-positive women is to prevent transmission. Once transmission has been prevented, the programme considers itself to be successful and usually ends. Programme failure to cast a gaze beyond its immediate disease-specific aim has a number of consequences. One recent example of this is the emerging evidence of increased mortality and morbidity among HIV-exposed, uninfected infants and children [48]. A broader focus on wellness within a family-based approach would reduce the potential for the broader implications of HIV infection (not simply transmission) to be overlooked.

Another example of the limitations of a disease-focused intervention from the parental depression literature is the finding of Seifer and colleagues [49] that poor parenting practices associated with depression may persist following a depressive episode and when the parent is relatively symptom free. This provides further evidence for a broader programme focus, rather than simply focusing on the depression [43].

A focus on early parenting that has characterized a number of interventions in the parental depression field has important lessons for HIV treatment and prevention. Punitive and coercive parenting has been associated with externalizing behaviour in children: children who exhibit these behaviours are more likely to get into trouble at school [50], have an earlier sexual debut [51], and engage in risky sexual behaviour [52], factors that are likely to increase the risk for HIV infection. Benefits of parent responsiveness-focused interventions have also been shown to extend to other areas of child health, including physical growth [53].

It has also been shown how a family-based approach impacts health, quality of life, and compliance with treatment regimens among HIV-positive parents [54]. Parental support and close family relationships are associated with later sexual initiation and increased condom use [55,56], while family cohesion and support are related to less risky sexual behaviour and fewer health-risk behaviours [57,58].

### A generational and developmental approach

In the light of the compelling evidence of the effects of depression on parenting skills and consequent child health and development, it is crucial that interventions are developed taking into account developmental stages of children, as well as using a generational approach. A two-generational approach (parent and child) or three-generational approach (grand parent-parent-child), together with a focus on siblings, immediately embeds any intervention in a broader familial-ecological context [59]. A family-based approach is, at its core, a generational approach. In the conventional understanding of the term, it is generational by virtue of the fact that it includes parents, children, siblings and grandparents.

In the context of maternal depression, the presence of other involved caregivers (father, grandparent, aunt or other) mitigates the impact of the maternal depression on the infant and child [9]. In the case of HIV, an individualized focus often ignores the significant familial barriers to, for example, exclusive breastfeeding driven by cultural and generational (mother-in-law, grandmother)prescriptions about appropriate infant feeding [60]. Unless significant family members, such as elders or mothers-in-law, "buy into" the notion of exclusive breastfeeding, it is highly unlikely that the decision to exclusively breastfeed(no matter how well intentioned) will find sufficient support within the family context to be successful.

In another understanding of a generational approach, family-based approaches (to depression or to HIV) are generational in that they have the potential to improve the context of children born into households at risk, and in so doing, improve long-term infant and child outcome. This form of intervention will reduce the likelihood of children engaging in risky behaviour across their life spans. A parenting intervention with parents and grand-parents aimed at improving monitoring of young children and facilitating less permissive parenting has been shown to be associated with adolescents having fewer sexual partners and fewer pregnancies [61]. The evidence presented here on the moderating effect of other (non-depressed) family members in the context of maternal depression further strengthens the argument for a generational approach.

Parental depression that occurs during infancy, upon the transition to school, or during adolescence has particular developmental implications that may be different from parental depression occurring at other developmental points. This is also the case with HIV, most pertinently, of course, in the context of mother to child transmission,, but it is also true at other stages of development. Financial constraints resulting in children not enrolling in school, or the implications of food insecurity for childhood stunting and malnutrition are two common examples. A family-based approach is "developmental" to the extent that it acknowledges how particular developmental milestones may throw up particular challenges to families, which may then require an intervention specifically tailored to fit the particular developmental stage of the child. Such sensitivity is difficult to incorporate when the focus is on the individual, and a narrow conception of disease.

### Family-based interventions

Weissbourd [62] has outlined four principles of family interventions that are pertinent to this discussion. The first principle is that there is no such thing as a child without a family, and that families only exist in the larger context of community life. The second principle is based on the evidence that families are better able to support themselves when they receive appropriate support; this is known as the family self-sufficiency model. The third principle is that it is cost effective and appropriate to foster positive and favourable development, rather than to merely avoid problems. The final principle is the recognition of the importance of the early years for infant and child development, and that in terms of brain development, it is through relationships with other people that synaptic connections are formed. Broad family-based interventions to mitigate the impact of parental depression usually comprise all or most of these four elements.

A focus on the family in no way excludes a focus on the health system or disease-specific strategies. What it does do, however, is include in programme design an understanding of how any health issue is firmly embedded within a familial context. In the case of infant feeding, for example, it acknowledges that simply providing information about exclusive and appropriate feeding, and even convincing HIV-positive women about it, is simply the first step in a complex chain of familial negotiations that will have to take place for the knowledge to become translated into practice. Interventions must address the environmental barriers to implementation.

Siblings constitute an important aspect of the family environment that is seldom considered. Positive sibling relationships can be protective for children exposed to stressors, especially in homes characterized by parental conflict [63,64]. When designing interventions, it is important that consideration be given to strengthening relationships between siblings with a view to reducing the effects of adverse experiences [63]. With the increasing occurrence of child-headed households, implementing preventive family-based interventions that target siblings from the outset is vital.

Given the cost of treating depression, and the lack of access to mental health care and psychotropic medication because of weak health systems in many low- and middle-income countries, an important consideration is the role of alternative caregivers [33]. There is evidence that infants of depressed mothers respond positively during interactions with their non-depressed fathers [65], as well as other caregivers, such as child minders or day-care nurses [66].

Interestingly, Cohn and colleagues [67] found a positive benefit for the mother-infant relationship when the depressed mother was not based at home full time. Alternative care has also been shown to reduce behaviour problems in children, aged two and three years, of depressed mothers [68]. These data are highly pertinent for HIV in that they illustrate how the functioning of other family members is central for beneficial child outcomes (even in the context of maternal depression).

## Discussion

Rotheram-Borus and her colleagues [35] have argued that a paradigm shift is needed in HIV prevention, treatment and care. The lack of skilled staff, poorly developed health systems and financial constraints all make the continuing focus on categorical funding (disease specific) ineffective [35]. Categorically funded, vertically integrated HIV interventions are highly stigmatized and will not have the capacity to address the health needs of Africa [35]. This is also true for depression, and unless packages of care for depression or other mental disorders [69] are integrated into community- and family-based intervention models, they are unlikely to be successfully implemented at scale.

While family-level interventions offer the potential for significant gains in the prevention and treatment of HIV, their implementation will face many of the same barriers that individual-focused interventions do. Scaling up family-based interventions will need to be linked to existing service delivery systems and integrated with the existing health care system. In addition, they will require a trained, well-managed and adequately supported workforce in order to deliver the interventions.

In the context of the significant human resource crisis that characterizes many low- and middle-income countries [70], community health workers are increasingly being used to deliver interventions. There are, however, significant barriers to the effective deployment of community health workers (such as training, monitoring and supervision). Another option to scaling up services that has met with some success has been to make use of the least costly health workers who are able to complete the task, otherwise known as task shifting [71]. A successful example of task shifting has been the use of surgically trained assistant medical officers to perform caesarian sections [72]. Recently, however, it has been argued that task shifting should not be seen as a panacea for the human resources challenges faced by low- and middle-income countries [73].

Depression and HIV are both highly stigmatized conditions. Furthermore, they are both chronic illnesses with repercussions for family members that go beyond the individuals and their illness. As a result, a family-focused wellness perspective is likely to be a more acceptable vehicle of intervention than a focus on any single condition or disease entity. Models of intervention focusing on early parenting, familial cohesion, illness detection and appropriate health-seeking behaviour, cognitive-behavioural strategies of behaviour change, linking people to poverty alleviation programmes, and comprehensive strategies that begin early in life and continue over time (characteristic of many successful intervention programmes in the domain of youth violence [74]) are urgently needed.

The broad diffusion of these successful programmes has not happened in any significant way [35]. There are many reasons for this, not least of which is the continuing search for the "magic bullet" for HIV prevention. One of the reasons for poor diffusion is that delivering efficacious treatments under ideal conditions is quite different from implementation at scale in community settings. Interventions are embedded within the "messiness" of family life, the chaos of families without meaningful routines, and with multiple familial actors that all contribute to both the problem and its solution. Behavioural change can only be sustained when it is supported by the routines and personal relationships that characterize daily family life[35]. This is simply not possible in individual-focused, disease-targeted interventions.

All disease-specific (or individual-focused) interventions are, to a greater or lesser degree, targeted responses. Stand-alone, single disease focused interventions for depression or HIV remain narrow in focus and are unlikely to impact meaningfully on child outcomes. So while the response to HIV is not like the mass eradication programmes characteristic of polio eradication or child health days (vitamin A supplementation, de-worming), the underlying focus is still on a specific disease.

The evidence from parental depression offers insights into how a shift from viewing HIV or depression as the primary focus, together with a family-based approach, allows us to "see" with greater clarity the extent to which these are embedded in contexts characterized by inter-personal violence, poor child attendance at school, absent fathers, chaotic family routines, intergenerational transmission of trauma, mental illness, youth violence and risk taking, and disempowerment of women.

Any move to a family-centred approach in poor countries will need a parallel development of a research agenda. The advantage of an individualized, disease-targeted approach is that measures of efficacy/effectiveness are often single outcomes linked to a single, (relatively) easily measured intervention (de-worming, vitamin A supplementation). Family-centred approaches, on the other hand, involve complex interactions between many levels of intervention and with multiple outcomes. Measurement is complex and this needs to be factored in when implementing and measuring family-based interventions.

## Conclusions

The aim of this paper has not been to set up individual, disease-targeted programmes in opposition to family-centred interventions. There is a place for both. It would be a mistake to now assume that family-based interventions are the next "magic bullet". I would argue, however, that the focus on individual, disease-focused interventions has tended to neglect the reality of how people are always embedded within families and broader communities, which has resulted (certainly in the case of depression and HIV) in an overemphasis on finding the magic bullet.

In the case of HIV, each and every magic bullet has failed [35] and shown to be hopelessly optimistic. Wagner and Blower [75] have shown, for example, how the latest magic bullet, the test-and-treat strategy that the WHO has argued would eliminate HIV within 10 years [76], is likely to be ineffective, and that even under optimistic conditions, HIV elimination using the test-and-treat strategy is (theoretically) possible only in 70 years' time.

The treatment and prevention of HIV requires, just as parental mental illness does, a multigenerational, develop mentally appropriate and integrated family-centred approach. Unless this is done, the fruitless search for the next magic bullet will continue unchecked.

## Competing interests

The author declares that they have no competing interests.

## Authors' contributions

MT drafted the manuscript. The author has approved the final manuscript.
